# Case Report: Indocyanine Green-Based Angiography for Real-Time Assessment of Superficial Brachialis Axial Pattern Flap Vascularization in Two Dogs

**DOI:** 10.3389/fvets.2022.859875

**Published:** 2022-04-18

**Authors:** David Michalik, Mirja Christine Nolff

**Affiliations:** Clinic for Small Animal Surgery, Vetsuisse Faculty, University of Zurich, Zurich, Switzerland

**Keywords:** near infrared angiography, NIRA, Indocyanine Green, ICG, axial pattern flaps, superficial brachial, skin reconstruction

## Abstract

This case report describes the method and feasibility of near-infrared angiography (NIRA) to improve the surgical procedure of two superficial brachial axial pattern flaps intraoperatively using two camera systems. Two client-owned dogs were treated for tumors on their antebrachia with wide surgical excision. The defects were closed with a superficial brachial flap in both cases. A different NIRA camera system was used for each case to identify the perforator vessel and flap margins accordingly. Case 1 developed a seroma and healed without further complications. Case 2 developed partial flap necrosis, underwent revision surgery, and healed by secondary intent. NIRA proved useful intraoperatively in identifying the perforator vessel and determining flap margins. As these are only two cases, caution should be used in extrapolating the results.

## Introduction

Axial pattern flaps (APFs) are used to cover big skin defects after traumatic skin loss or oncologic surgery in veterinary medicine. Complication rates of APFs in dogs and cats have been reported to be up to 89% (1, 2). Among the complications, necrosis is one of the most threatening (21–46%), as it frequently leads to skin loss and, ultimately, flap failure. Different reasons were stated to contribute to necrosis, including surgical technique, or misjudgment of the dermatome or the perforator vessel location, resulting in trauma to the flap during or after preparation (3–5).

Near-infrared angiography (NIRA) is a technique that allows for real-time visualization of the vasculature during surgery. A cyanine dye like Indocyanine Green (ICG) is given intravenously. It is visualized in the near-infrared (NIR) spectrum with a corresponding imaging system. In human reconstructive surgery, NIRA using ICG is frequently performed to assess the perforator vessel and skin vascularization, and determine the outline of the flap (6–9). NIRA-based reconstruction has a strong correlation with positive outcomes when performed for pedicle perforator flaps or large skin paddles (10, 11).

In veterinary medicine, NIRA using ICG in skin reconstructive surgery has only been described in two cats for visualization of auricular oris pattern flaps (12). The authors found the technique useful, and flap outcome was excellent in both cases.

NIRA could also prove useful to evaluate flap perfusion in dogs. The following case report describes the application and possible limitations of ICG NIRA with two clinical imaging systems in reconstruction using the superficial brachial APF in two client-owned dogs.

## Case Descriptions

### Case No. 1

**Figure 1:**
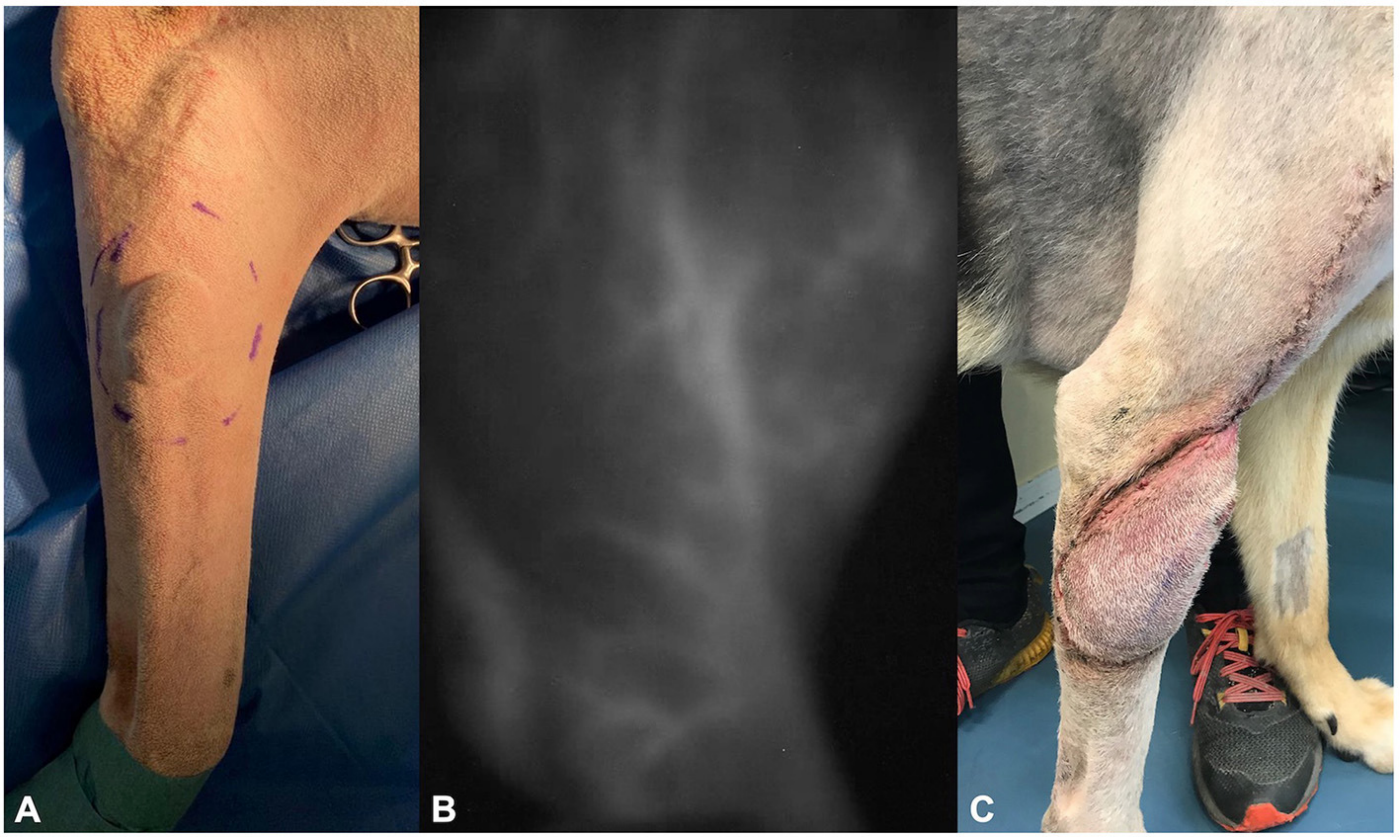
Photographs of dog 1, showing **(A)** the soft tissue sarcoma preoperatively, **(B)** the delineation of the superficial brachial artery using the IC-Flow™ camera system intraoperatively, and **(C)** flap edema at day 20 postoperatively.

A 6.5-year-old male castrated mixed-breed dog (38 kg) was presented due to a subcutaneous soft tissue mass on the right latero-proximal antebrachium, which had been noted by the owner 6 weeks prior to presentation and was rapidly growing (Figure 1A).

Fine-needle aspiration was performed by the referring veterinarian. Cytology showed high suspicion of soft tissue sarcoma. Radiographs of the thorax, cytology of the superficial cervical lymph node, complete blood cell count (CBC), and blood chemistry were normal. Because of the suspected sarcoma, options for treatment (including marginal resection followed by radiotherapy vs. surgery with curative intend, 3-cm margins, and one fascial plane beneath the tumor) were discussed with the owners who opted for surgery with curative intent. Based on the location of the mass, reconstruction using a superficial brachial axial pattern flap was considered best reconstructive approach for this case.

### Surgical Procedure

After following a standard premedication protocol and anesthesia induction, the dog was positioned in left lateral recumbency with the right front leg in hanging leg position.

The tumor was removed with 3-cm lateral margins and the fascia of the common and lateral digital extensor muscles as a deep margin. The resection site was then covered with moist gauze, and instruments and surgical gloves were replaced. NIRA was then performed using the IC-Flow camera system (IC-Flow™; Diagnostic Green GmbH), which cannot be operated under ambient light. Ambient and OP lights were turned off, and 0.5 mg/kg ICG (Verdye Diagnostic Green® 1.25 mg/ml; Diagnostic Green GmbH) was injected as a bolus into the brachiocephalic vein of the contralateral leg. The near infrared camera was turned on, and within 65 s after injection of the ICG-dye, the superficial brachial artery was fully visible (Figure 1B). The required length of the axial pattern flap was measured with a ruler and marked with a sterile skin marker. The flap was then lifted from proximal to distal, mobilized, and rotated to the defect on the latero-proximal antebrachium. Both wounds were splashed with ropivacaine 2 mg/kg (Ropivacain Sintetica® Sintetica SA). The vascularization was controlled *via* the NIR camera, and the flap was sutured into the defect using simple interrupted sutures followed by a continuous intracutaneous suture Polydioxanone 2.0 (Monoplus®, Braun GmbH).

Surgery time was 75 min. Following completion of the surgery, a bandage incorporating the shoulder was applied. The dog recovered well from the anesthesia and was discharged the day after surgery. Postoperative analgesia was continued using robenacoxib 2 mg/kg (Onsior® Elanco Tiergesundheit AG) injected subcutaneously at the end of the surgery and followed by 1 mg/kg orally once daily for 4 days.

### Follow-Up

The bandage was removed by the owner 6 days after the surgery. Clinical follow-ups were done on days 6 and 13 post-surgery. Seroma formation was observed at first re-check and treated with warm packing three times daily until regression (Figure 1C). No further complications occurred. Histopathology showed hemangiopericytoma grade 1, which was completely resected (R0, all lateral margins > 5 mm, deep margin 1 fascial plane, non-invaded). Based on these results, no further adjuvant therapy was prescribed. The dog had no signs of tumor recurrence or flap-related complications 12 months post operation.

### Case No. 2

**Figure 2:**
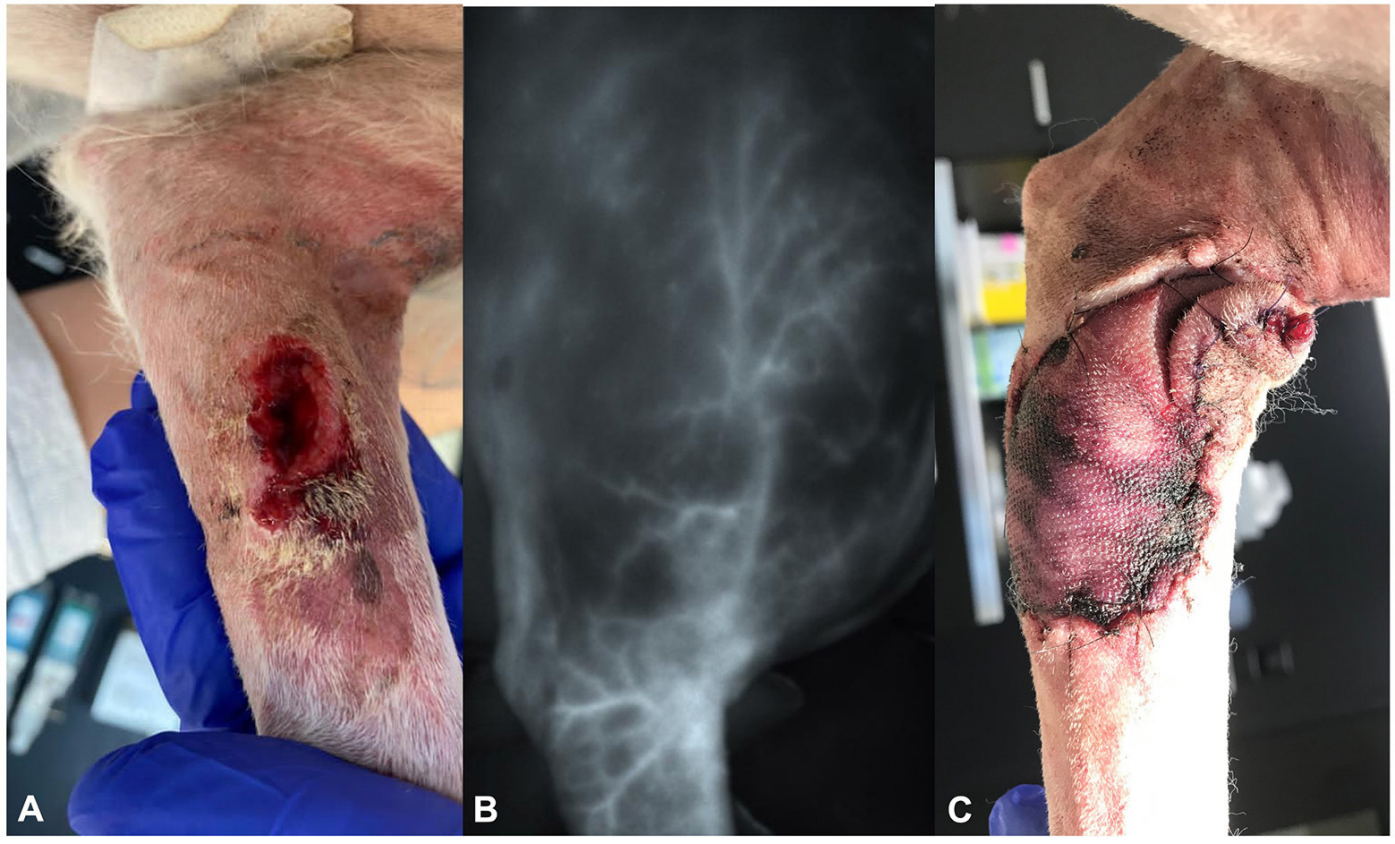
Photographs of dog 2, showing **(A)** the dehiscent wound on the left medio-proximal antebrachium at presentation, **(B)** the delineation of the superficial brachial artery using the VisionSense VS3-IR-MMS camera system intraoperatively, and **(C)** the discoloration of the distal edges of the flap on day 6 postoperatively.

An 8-year-old mixed-breed dog (13.5 kg) was presented with a wound infection and partial skin necrosis 6 days after debulking surgery of a 2.5 cm subcutaneous mass on the left medio-proximal forearm by the referring veterinarian. No pre-surgical diagnosis of the mass was obtained. Histology revealed an incompletely excised mast cell tumor (Patnaik grade II, Kiupel low grade with MI <1). After the surgery, the dog developed a wound infection and partial skin necrosis at the surgical site and was already under amoxicillin-clavulanic acid 20 mg/kg twice daily (Clavaseptin® Vetoquinol AG) and carprofen 4 mg/kg once daily (Canidryl flavor 100 mg; Dr. E. Graeub AG). It was then transferred for wound management and further treatment of the incompletely resected mast cell tumor (Figure 2A).

At presentation, the physical examination additionally revealed a mildly enlarged superficial cervical lymph node. Fine-needle aspiration was conducted and showed no signs of metastasis. Hematology showed mild leukocytosis (15 × 10^9^/l). Further staging was not performed because of the owner's request.

The wound was treated locally until signs of the infection had resolved. The dog was then represented for revision of the tumor site and lymphadenectomy of the sentinel lymph node 10 days after the initial presentation.

### Surgical Procedure

**Figure 3:**
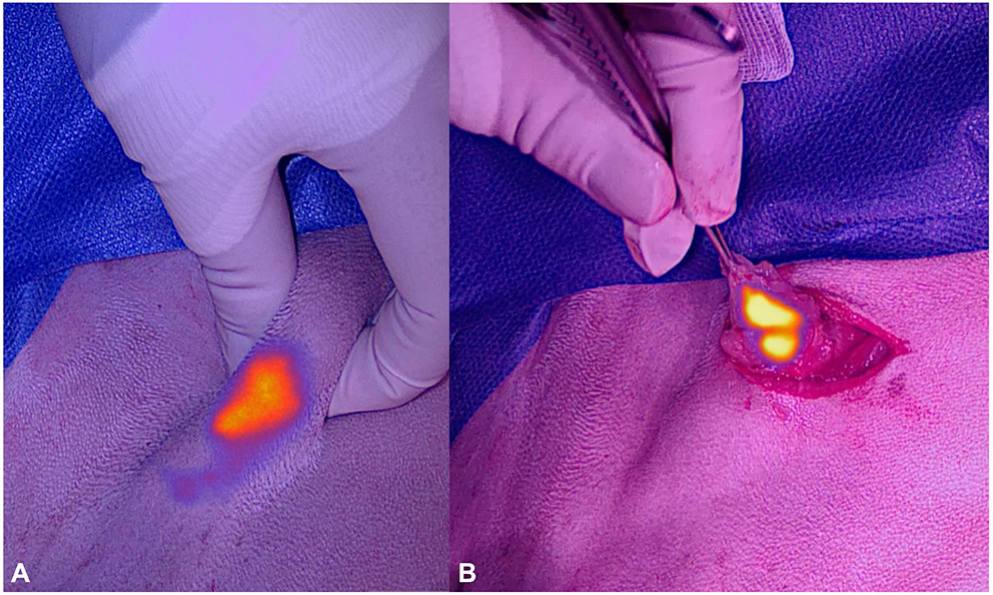
Photographs of the near-infrared signal of the superficial cervical lymph node of dog 2 before **(A)** and during excision **(B)** under ambient light.

As the wound was not infected but considered contaminated, amoxicillin clavulanic acid was restarted before surgery at a dose of 20 mg/kg to avoid flap infection. After following a standard premedication protocol and anesthesia induction, the dog was placed in right lateral recumbency, and ICG-based lymphography was performed to identify the sentinel lymph node. One milliliter of 1.25 mg/ml ICG was injected into the skin at four quadrants surrounding wound edges. Lymphatics were then visualized using the VisionSense VS3-IR-MMS™ camera system [Medtronic AG (Schweiz)] in 3DHD lymphography setting (working distance 30 cm, 90°angle). Contrary to the system used in case 1, this system offers true real time NIR imaging, as it can be operated under ambient and surgical lights. The left superficial cervical lymph node was identified transcutaneously as the draining lymph node (Figure 3A). A 2 cm incision was made directly overlying the NIR signal of the node, and the node was excised after separation of the muscle fibers of the brachiocephalic muscle (Figure 3B). Recorded time for the lymph node resection was 4 min and 58 s. The wound was then closed routinely using a 2.0 glycolate suture (2.0 Monosyn® Braun GmbH). Total time for the lymphography and lymphadenectomy was 11 min.

The wound from the first tumor removal was then excised en-bloc with 2 cm lateral margins, including the fascia of the superficial digital flexor muscle as deep margin and covered with a moist swab. ICG 0.5 mg/kg was injected intravenously as described in case 1. The VisionSense VS3-IR-MMS™ camera system was operated in angiography setting (30-cm working distance, 90° angle) and used to map the course of the superficial brachial artery (Figure 2B). The recorded time from IV injection to clear visualization of the vessel was 93 s.

The outline of the flap was marked with a sterile skin marker. The flap was developed as described before, and then mobilized and rotated approximately 180° to the defect on the medio-proximal antebrachium. Vascularization was controlled with the near infrared camera, and the flap was sutured into the defect. A bandage incorporating the shoulder was applied. Surgery time was 89 min.

The dog recovered well from the anesthesia and was discharged the same day with cage rest and an Elizabethan collar. Analgesia was maintained by prescription of carprofen (4 mg/kg once daily (Canidryl flavor 100 mg; Dr. E. Graeub AG), and amoxicillin-clavulanic acid was continued at 20 mg/kg three times daily.

### Follow-Up

The histology report showed complete (R0) resection of the grade II (Patnaik)/low-grade (Kiupil) mast cell tumor with a metastatic lymph node classified as HN2 (based on Weishaar classification).

Three and 6 days postoperatively, the flap showed no sign of edema or seroma; however, the craniodistal and caudodistal borders showed dark red discoloration but had no signs of induration or temperature differences compared to the surrounding tissues (Figure 2C). A new bandage was reapplied to avoid edema formation and improve microcirculation. As there were no signs of infection and the mast cell tumor was completely resected, amoxicillin-clavulanic acid and cetirizine were discontinued at this point.

After another 3 days, the flap developed partial necrosis involving approximately 50% of the total flap length.

The necrotic area was then excised 12 days after reconstruction, and the wound was treated by open wound management. Complete wound healing was achieved 33 days after debridement. Up until the time of writing (11 months after complete healing), the dog still had not developed any further complications or tumor recurrence.

## Discussion

These cases report the successful application of ICG-based NIRA for identification of the superficial brachial artery before flap transfer in two dogs. The superficial brachial artery and vein are reportedly hard to identify and are at risk of being damaged during flap dissection (13). Distinct delineation of the superficial brachial artery after intravenous injection of 0.5mg/kg ICG dye was successfully achieved with both camera systems within 75 and 93 s after injection and helped to guide the surgeon in identifying the APF-margins.

Intravenous injection of ICG has been previously determined to be safe for dogs (14, 15). Our findings underline this, as no adverse effects were noticed in conjunction with the injection (including no alterations in heart rate or blood pressure).

General anatomical landmarks for development of the superficial brachialis flap have been described as follows: “*the flap base includes the flexor surface of elbow, anterior and posterior borders lateral and medial parallel to the humeral shaft approaching the greater tubercle:* (16, 17).”

**Figure 4:**
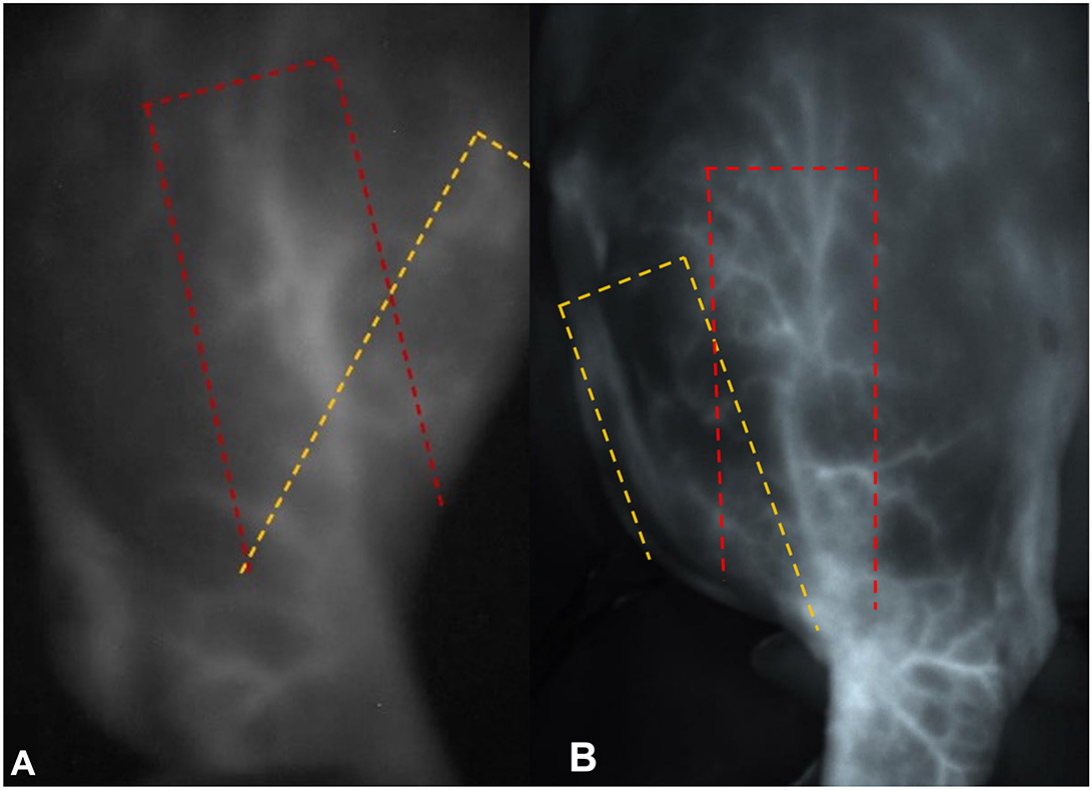
Photographs of the near infrared signal of the superficial brachial artery in **(A)** dog 1 and **(B)** dog 2 (under ambient light). In both pictures the yellow dashed line shows the theoretical anatomical landmarks of the flap, while the red dashed line shows the individual course of the vessel.

In both of our cases, the vessel presented more laterally than anticipated by the surgeon based on the accepted flap outlines (Figures 4A,B). Reasons for this might be individual variation and impact of the positioning of the patient intraoperatively. We consider individual variation to be the most likely explanation; however, the course of the vessel was consistent in both patients. Further *in vivo* angiography studies will be needed to evaluate if these are truly patient-specific alterations or if the course of this vessel is more lateral than previously described.

Two NIR camera systems were used in the described cases. The IC-Flow™ camera used on dog 1 is being used in this university for 3 years now mainly for NIR lymphography. It is a portable, easy to use, handheld system. There is a learning curve, and the handheld version requires an assistant to hold the camera in place, resulting in considerable impairment of vision due to imperfect focus and movement artifacts (Figure 1B). In addition, this system cannot be used under ambient light during surgery. This means NIRA can only be performed in complete darkness in an operating room, adding in procedure time as NIR imaging and live images need to be repeatedly compared during surgery. This also precludes real-time imaging, as a surgeon must memorize the image and transfer it to the situation while performing the surgery with the lights on. Although less susceptible to inaccuracy, an alternative would be to mark the perforator vessel with a skin marker while NIRA is being performed.

Same limitations were also described by Quinlan et al. (12) in using the VITOM II ICG exoscope on two auricularis oris flaps in cats. Inability to operate under ambient light is challenging for a surgeon, because anatomical structures are not clearly seen while performing (ICG-Angiography), compromising the safety and accuracy of the procedure.

Between cases, the imaging system was changed to the VisionSense VS3-IR-MMS system. The imager offers different imaging settings with quantification of signal intensities, real-time overlays, and imaging under ambient light. In a recent comparison of various clinical imager systems performed by DSouza et al. (18), the system was rated as one of the leading edge imaging systems with the following specifications: real-time white light and fluorescence overlay possible, good quantification linearity, and working distance of 30 cm.

We found this system very easy to use because of the mounted camera, option to perform surgery without any impairment from lighting, and excellent image quality (Figure 2B). In addition, the various settings allow for the adaptation of the system to fluorophore intensity. Subjectively, these features make ICGA (ICG-Angiography) very intuitive to use and provide excellent vision of the region and vessel of interest. However, as already discovered in lymphography before, there is a substantial learning curve that must be anticipated when starting NIRA.

NIRA performed with either system resulted in clear delineation and identification of the vessel during surgery. The recorded time for clearing the delineation of the vessel was 75 and 93 s, which was slightly longer than the time recorded by Quinlan et al. (12) for NIRA in visualization of auricularis flaps in cats (35 and 52 s). This might be due to species differences, including weight and heart rate at the time of injection. Another explanation would be difference in sequential visualization of different dermatomes. Additional studies will be needed to understand these differences. Nevertheless, NIRA can be performed rapidly after injection. This point is important, as no substantial delays in surgery time should be expected when using the technique.

Despite the improved surgical technique, dog 2 developed a complication directly associated with impaired perfusion, with necrosis in a large portion of the flap. Partial flap necrosis of APFs is described to be as frequent as 20% in dogs (2). A recent study by Villedieu et al. (19) reported 100% complication rate in superficial brachial flaps, with flap necrosis in 7 out of 16 cases. Different theories for this have been proposed, but the reasons remain unclear. The relatively small size of the perforator vessel may play a critical role in reduced vascularization after preparation and transposition. Another factor influencing flap perfusion directly after translation could be posture of the leg. After transposition, the perforator vessel runs directly over the elbow joint. Repeated flexion of the joint could provoke early kinking, causing edema and malperfusion. To avoid seroma formation and excessive movement, a bandage was placed in both cases postoperatively for 6 days. Additional cast immobilization for a short post-operative period could have mitigated flexion of the elbow even more and, therefore, could have reduced the chance of developing mal-perfusion.

After rotation of the flap into the defect intraoperatively, neither the IC-Flow™ nor the Visionsense 3D showed comparable vessel delineation to the pre-transposition signal of the superficial brachialis artery. This could indicate trauma during preparation or kinking of the vessel after rotation, resulting in compromised blood supply. As this was seen in both dogs but only one developed necrosis, it is unclear how important this finding is. Preparation of flaps, including a deep layer of additional tissue or muscle, might help to reduce direct trauma to the vasculature, but this must also be elucidated in further studies. Other reasons for loss of signal after transposition could be the fast plasma clearance rate of ICG in anesthetized dogs, 30.6 ± 8.3 ml/kg/min, correlating to an ICG plasma disappearance rate (ICG-PDR) of 7.4 ± 2.8%/min (20). That means after 7 min <50% of ICG is still circulating in the plasma. In human medicine, plasma clearance rate is even faster and considered one of the biggest limitations in ICG-based NIRA. The initial half-life in humans is 3–4 min with a dose of 0.5 mg/kg, making it difficult to assess perforator vessels for a skin flap intraoperatively without reinjecting (21).

A second injection of ICG intraoperatively after flap transposition should be strongly considered to detect changes in circulation after flap transposition, such as vascular kinking, thrombosis, and local vasospasms after manipulation. Not performing a reinjection of ICG post flap translation in both cases poses a limitation in the described technique, as it is hard to evaluate if the ICG signal is lost because of higher plasma disappearance or other reasons. In human medicine, the impact of ICG-based NIRA on outcome has been controversially discussed.

ICG-based NIRA is accepted as a beneficial tool for intraoperative decisions in reconstructive surgery (22, 23). Further studies have shown that usage of this technique decreases the amount of flap complications in pedicle flaps (11, 24).

However, based on a recent meta-analysis, Pruimboom et al. (25) stated that it is not possible to draw a clear conclusion on how perfusion imaging really correlates with outcome. This is because exact ICG dosing, camera angle, and working distance are frequently not standardized across and within studies. As these all impact the intensity of the detected signal, future studies need to include these parameters to improve the predictive value of ICG-based NIRA. Prospective controlled studies are unavailable on human and veterinary medicine.

## Conclusion

ICG-based NIRA is a safe, easy, and fast technique for identifying the superficial brachialis artery in dogs intraoperatively. Although the authors consider this very helpful during surgery, this case description does not allow for the evaluation of the clinical impact of the technique on complication rates.

As ICG adds additional costs to the procedure (depending on the country) and access to a NIRA camera must be warranted, it is important to evaluate if ICG-based NIRA in reconstruction surgery is worth the clinical benefit.

## Data Availability Statement

The raw data supporting the conclusions of this article will be made available by the authors, without undue reservation.

## Ethics Statement

Ethical review and approval was not required for the animal study because the use of intravenous ICG and the use of a NIRA camera are accepted methods and therefore do not need an Animal Ethics Committee. Written informed consent was obtained from the owners for the participation of their animals in this study.

## Author Contributions

DM and MN contributed to conception and design of this case report. DM wrote the draft of the manuscript. MN edited the manuscript and oversaw the project. Both authors contributed to manuscript revision, read, and approved the submitted version.

## Conflict of Interest

The authors declare that the research was conducted in the absence of any commercial or financial relationships that could be construed as a potential conflict of interest.

## Publisher's Note

All claims expressed in this article are solely those of the authors and do not necessarily represent those of their affiliated organizations, or those of the publisher, the editors and the reviewers. Any product that may be evaluated in this article, or claim that may be made by its manufacturer, is not guaranteed or endorsed by the publisher.

## Supplementary Material

Supplementary Video 1 The Video shows the delineated left superficial brachial artery intra operatively after intravenous ICG injection in Dog 2 in the color fused image. A skin marker is used to indicate the course of the vessel to facilitate flap margins in the next step.
